# SpyShield: a Spyfall inspired defense mechanism against poisoning attacks in federated learning

**DOI:** 10.1038/s41598-025-16158-3

**Published:** 2025-08-26

**Authors:** Youssef Elgharieb, Wassim Alexan, Minar El-Aasser, Milad Michel Ghantous

**Affiliations:** 1 CSEN Department, Faculty of MET, German University in Cairo, Cairo, Egypt; 2 Communications Department, Faculty of IET, German University in Cairo, Cairo, Egypt; 3 Networking Department, Faculty of IET, German University in Cairo, Cairo, Egypt; 4 DMET Department, Faculty of MET, German University in Cairo, Cairo, Egypt

**Keywords:** Aggregation, Federated learning, Malicious clients, Poisoning attack, Poisoning defense, Engineering, Mathematics and computing

## Abstract

Traditional machine learning (ML) relies on a centralized architecture, which makes it unsuitable for applications where data privacy is critical. Federated Learning (FL) addresses this issue by allowing multiple parties to collaboratively train models without sharing their raw data. However, FL is susceptible to data and model poisoning attacks that can severely disrupt the learning process. Existing literature indicates that defense mechanisms predominantly analyze client updates on the server side, often without requiring or involving client cooperation. This paper proposes a novel defense mechanism, SpyShield, that leverages client cooperation to identify malicious clients in data and model poisoning attacks. SpyShield is inspired by tactics used in the social deduction game Spyfall, where the majority of players must detect the deception of a minority, a dynamic aligning with the challenges posed by poisoning attacks in ML. In this paper, we evaluate four different configurations of SpyShield’s robustness and performance on the FashionMNIST dataset against five benchmark aggregation algorithms–FedAvg, Krum, Multi-Krum, Median, and Trimmed Mean–under three attack types: (A) Cyclic Label Flipping, (B) Random Label Flipping, and (C) Random Weight Attacks. Each attack is tested across three scenarios: (I) 3 malicious clients out of 30, (II) 10 out of 50, and (III) 40 out of 100, totaling nine experimental settings. These settings simulate varying attack intensities, allowing the assessment of SpyShield’s effectiveness under different attack invasiveness. In every setting, at least one configuration of SpyShield consistently outperformed all benchmark algorithms, achieving the highest accuracy. The evaluation shows that SpyShield achieves strong performance and resilience across diverse settings and attack types. These findings highlight its potential as a robust and generalizable defense mechanism for securing federated learning models, while also opening new possibilities for collaborative strategies that move beyond centralized server-side analysis.

## Introduction

Traditional ML faces a major problem, which is privacy. The centralized nature of ML methods is naturally invasive of users’ privacy, as it requires the data to be stored in a central location^1^, making it susceptible to exploitation. Even if the data is anonymized, it can still be traced back to its source^2^. Thus, traditional ML is ill-suited to privacy-sensitive fields such as health care^3^ and next-word prediction^4^. A new breed of distributed ML, i.e., FL, solves this problem as it ”brings the code to the data, instead of the data to the code,” as stated in^5^.

Federated learning presents multiple security and privacy challenges^6^ that remain active research areas^7^. One of the primary security concerns is data and model poisoning, where one or more client devices send malicious updates that compromise the global model. This attack is very destructive in FL, as even a small number of malicious clients can render the contributions of other clients futile. Regarding privacy, the major concern is inference, where malicious agents can deduce a client’s data using model-inversion techniques.

To the best of our knowledge, all the current literature on defending against data poisoning is based solely on analyzing the clients’ updates on the server, as in^8^. However, it is worth considering collaborative approaches involving both the server and clients to identify and isolate adversaries. This article argues that a collaborative approach involving input from clients is effective in defending FL against poisoning attacks. In support of this argument, this article proposes *SpyShield*, a novel defense mechanism that harnesses the collective intelligence of clients to identify potential adversaries.

SpyShield draws on the concept of majority cooperation against minority deception from the game of Spyfall. In Spyfall, players collaborate to identify spies who are attempting to mislead the group. Similarly, in FL, the majority of clients are expected to contribute legitimate data, while a minority may harbor malicious intent. Modeling this challenge as a social deduction game of Spyfall offers a new approach to enhancing the robustness of FL systems against poisoning attacks.

The contributions of this work are as follows:

1. Proposing SpyShield, a novel aggregation technique that defends against both model and data poisoning in an FL environment using clients’ knowledge to identify the malicious clients among themselves, mimicking the players’ behavior in the social deduction game Spyfall.
2. Conducting FL training simulations on the FashionMNIST dataset to evaluate SpyShield’s robustness under three attack scenarios: (A) Cyclic Label Flipping, (B) Random Label Flipping, and (C) Random Weight Attacks. These simulations were performed with varying malicious client ratios of (I) 3 out of 30, (II) 10 out of 50, and (III) 40 out of 100, totaling 84 simulations. Among these, 36 simulations employed SpyShield’s defense mechanism, while the remaining simulations employed benchmark defense mechanisms–FedAvg^5^, Krum, Multi-Krum^9^, Median, and Trimmed Mean^10^–to compare results and further validate SpyShield’s robustness.

This article is organized as follows. Section “Related work” discusses related work on poisoning attacks, their defense, and inference risks in FL. Section “Methodology” formally presents the methodology, outlining all the details of SpyShield. Section “Experiments” describes and discusses the experiments conducted on SpyShield and other benchmark algorithms, then presents the results, validating SpyShield’s robustness. Finally, Section “Conclusions” concludes this work and mentions possible future research directions.

## Related work

This section presents the various relevant concepts and related works. Subsection “Federated Learning” briefly introduces FL. Subsection  “Reconstruction Attacks” describes inference risks and mitigation mechanisms relevant to this work. Similarly, Subsection “Poisoning Attacks” describes the different types of poisoning attacks and current defense mechanisms in the literature.

### Federated learning

Federated learning was first introduced by Google in 2016^5^ for Google’s G Board keyboard prediction, query suggestions, and learning out-of-vocabulary words^4^. Federated learning represents a collaborative ML approach to solving some of the important limitations that exist within centralized ML approaches. In classic ML, data from various sources is aggregated and collected for model training, which has raised concerns about privacy, security, and the practicality of aggregating large volumes of data into a single location^11^. While data is increasingly being disseminated across multiple devices and institutions, especially in highly sensitive domains such as healthcare, finance, and IoT, the classical ML approach can no longer match efficiency with privacy.

Federated learning provided the above challenges by training ML models directly on decentralized devices or servers without having to share raw data. This approach enables each device, the so-called client, to train the model locally with its data never leaving the device. Only the client’s local model updates and not the data itself are sent to a central server for aggregation with other clients’ local models using FedAvg, an algorithm that computes a weighted average of local model updates to refine the global model while preserving privacy, hence keeping the data private and reducing risks related to data centralization. This enabled the adoption of AI-driven medical applications^12,13^. Moreover, FL reduces latency and bandwidth costs due to the transfer of huge datasets; hence, it is apt for applications where data is naturally distributed. Addressing these challenges, FL has been considered one major development in privacy-preserving, scalable, and efficient ML systems.

FL could be divided into two main types in terms of communication topography: a) central topology, in which a central server orchestrates the FL process, and b) fully decentralized peer-to-peer^14^ topology, where communication is carried out through peers directly. In this work, SpyShield defends FL against data and model poisoning in central topology. However, the defense mechanism could be adapted for a peer-to-peer topology in the future.

Despite this, FL faces several challenges. Mainly, two types of attacks pose a threat to FL^15^: reconstruction attacks^16,17^ and poisoning attacks^18^.

### Reconstruction attacks

Reconstruction attacks, also referred to as attribute inference or model inversion, are a significant concern in FL, as they involve adversaries attempting to reconstruct the original input data on which a local model was trained^19^. In a reconstruction attack, attackers exploit the model’s updated parameters, or gradients provided by clients before they are sent to the server, to infer private details about the data used to train the models. This is particularly problematic in FL because the data itself is never shared directly, but the information gleaned from model updates can still potentially reveal private information. For instance, attackers could use statistical analysis or ML techniques to reconstruct or infer characteristics of the training data, thereby compromising user privacy. Such attacks can be detrimental, especially in sensitive applications like healthcare or finance, where maintaining confidentiality is crucial. Existing solutions that mitigate the risk of reconstruction are the following:

1. Differential Privacy (DP)^20^: This was first introduced in 2006^21,22^ as a privacy-preserving mechanism that protects individual data points by adding controlled noise to sensitive datasets. This prevents adversaries from reconstructing the original data samples by limiting the impact of a single data point. In FL, noise is added to the client’s updated local model parameters before being sent to the server^23^. It balances privacy and accuracy, making FL more secure. Recent advances^24^ focus on increasing the robustness of DP in FL while minimizing the effect on accuracy.
2. Secure Aggregation (SA)^25^: These are protocols designed to protect individual clients’ model updates by ensuring that only the aggregated result is accessible to the central server, thereby preventing the server from inspecting individual contributions. This is achieved through cryptographic techniques that mask individual updates, which, when combined, the masks cancel out, revealing only the aggregated sum. Secure aggregation enhances privacy in FL by making it computationally infeasible for adversaries to reconstruct individual data points from the aggregated updates. However, recent studies have identified potential vulnerabilities in SA protocols. For instance, certain attacks can exploit predictable masking values to compromise user privacy, underscoring the need for dynamic and unpredictable masking strategies^26^.
3. Homomorphic Encryption (HE)^27^: This allows computations to be performed directly on encrypted data without requiring decryption, ensuring that raw data remains confidential throughout the process. In the context of FL, HE enables the central server to aggregate encrypted model updates from clients and perform necessary computations without accessing the underlying data. This approach provides strong privacy guarantees, as the server processes only encrypted information. However, implementing HE in FL presents challenges, primarily due to the significant computational and communication overhead associated with encrypting and decrypting data, as well as performing operations on encrypted data. To address these issues, integrating HE with other cryptographic methods, such as Secure Multi-Party Computation, has been explored to improve security against data reconstruction attacks in FL^28^.

### Poisoning attacks

Poisoning attacks target the integrity of the FL process itself. In poisoning attacks^29,30^, malicious clients deliberately manipulate their local data or model updates to corrupt the global model. There are two primary types of poisoning attacks:

1. Data Poisoning Attacks. These involve injecting malicious data into the training process. By submitting altered or intentionally misleading data, attackers can degrade the performance of the global model, leading to inaccurate predictions or decisions. This type of attack can be particularly challenging to detect and mitigate because the malicious data is blended with legitimate data, making it difficult to distinguish between harmful and harmless contributions^18,31^.
2. Model Poisoning Attacks. These occur when attackers send corrupted model updates to the central server. Here, the attacker alters the gradients or model parameters during the training process to influence the global model’s behavior in a harmful way. This could result in a model that performs poorly or behaves in an adversarial manner, which can be especially problematic if the model is used in critical decision-making systems^32,33^. Recent studies have also explored advanced attack strategies that combine elements of data and model poisoning, such as clean-label backdoors and universal impersonation^34–37^. While highly relevant, these hybrid attacks are beyond the scope of this work.

One of the defenses against poisoning attacks is robust aggregation methods that can tolerate and mitigate the impact of malicious updates. Techniques such as robust averaging, where the influence of outlier updates is minimized, or using statistical measures to identify and exclude anomalous updates, help ensure that the global model is less affected by poisoned data^38^.

Several notable robust aggregation methods are designed to counteract such attacks:

1. **Median and trimmed mean aggregation** methods focus on mitigating the impact of outliers or malicious updates by focusing on central tendencies^39^. The median aggregation method takes the median value of each model parameter from all client updates instead of averaging all client updates. This approach is robust to outliers because the median is less sensitive to extreme values^39^. The trimmed mean aggregation method involves discarding a fraction of the most extreme values (both high and low) and then averaging the remaining values. This reduces the influence of outliers on the aggregated result^40^.
2. **Krum** aggregation is designed to robustly handle the presence of a limited number of malicious clients by selecting updates that are most representative of the majority. It selects the update that minimizes the sum of distances to all other updates, assuming that the majority of the clients are honest. This method effectively filters out outlier updates that deviate significantly from the consensus^41^.

Aggregation methods such as Krum, median, and trimmed mean continue to struggle when applied to non-homogeneous (non-i.i.d.) datasets. More recent algorithms such as FLTrust^42^, rely on the presence of a trusted dataset at the server. FLTrust incorporates a base dataset to evaluate client updates using cosine similarity. However, this strategy depends on the unrealistic premise that the server has access to untainted data.

Similarly, some defense methods utilize structural insights beyond model updates to detect poisoned clients. For instance, the Multi-Party Immune System (MPIS)^43^ moves beyond traditional aggregation by incorporating client-reported data distribution information. MPIS validates client honesty by comparing claimed distributions with the actual updates, serving more as an authentication layer than a collaborative detection mechanism. MPIS introduces a novel idea but still relies on assumptions or forms of client-side data disclosure that may compromise privacy.

In contrast, the approach proposed in this article differs from existing literature by delegating the identification of malicious clients to the clients themselves, who possess greater domain knowledge needed that the server lacks in a realistic scenario. This work presents SpyShield, a comprehensive defense strategy against data and model poisoning attacks in FL, harnessing the clients’ collective knowledge.

## Methodology

In the reviewed literature on defending against poisoning attacks in FL, all approaches, including those in^9,43,44^, are implemented solely on the server without any assistance from clients. However, it is reasonable to collaborate with clients who are the first beneficiaries of defending against poisoning in FL to achieve this goal. This work proposes SpyShield, a new approach to defend against poisoning attacks in FL inspired by Spyfall, which is a social deduction game of game dynamics similar to that present in the challenge of defending FL against poisoning.

This section is divided into four subsections. The first subsection briefly explains Spyfall and the resemblance between it and the problem of identifying adversaries in FL. The three remaining subsections discuss the details of SpyShield’s implementation.

### Analogy

1) *Social deduction games:* These games involve players who are divided into two categories: an uninformed majority and an informed minority. The minority are informed which players belong to which group. The majority’s goal is to uncover the true identity of the minority members, while the minority aims to deceive the majority into thinking they are also part of the uninformed group. One of the oldest and most well-known examples is Mafia, a classic party game. Detecting poisoning in FL could be modeled as a social deduction game where the first uninformed minority is analogous to honest clients, and the second informed majority could be the set of adversaries.

2) *Spyfall:* Spyfall is a social deduction party game that shares striking similarities with the FL scenario. Unlike some other social deduction games, all the players are uninformed about who belongs to which group.

**Fig. 1:**
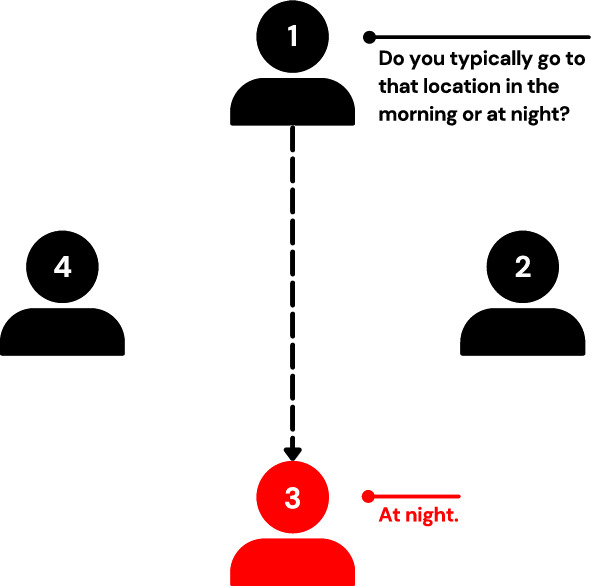
Visualization of the Spyfall gameplay, in which player (client) 3 is a spy, hence answering player (client) one’s question wrong.

The game begins with each player receiving a card face-down. The card either indicates a location (e.g., beach, nightclub, circus, museum) or is simply labeled ”Spy.” The majority of the players receive the cards with the same location, such as ”Beach,” while the minority receive the cards labeled ”Spy.” Since all cards are dealt face-down, none of the players know which group any other player belongs to. The majority aims to identify the spies, while the spies attempt to guess the location. Players then take turns asking each other questions to assess if the other is a spy without giving away the location. For example, if the location is the beach, one could ask, ”Do you typically go to that location in the morning or at night?” as demonstrated in Fig. 1.

After asking enough questions, before the timer runs out or the spies guess the location, players can vote on who they believe are the spies. All players get a vote, including the spies, meaning that spies actively participate in the discussion and influence other players’ votes. If the players voted on are indeed the spies, the majority group wins. If the players happen to vote off a client who is not a spy, the minority group of spies wins.

3) *SpyShield Question:* Analogously, in FL, we need to decide which clients, a minority, are malicious, analogous to the spies. The question that the server needs to ask a client to determine whether another client’s local model is poisoned is what this subsection discusses.

**Fig. 2:**
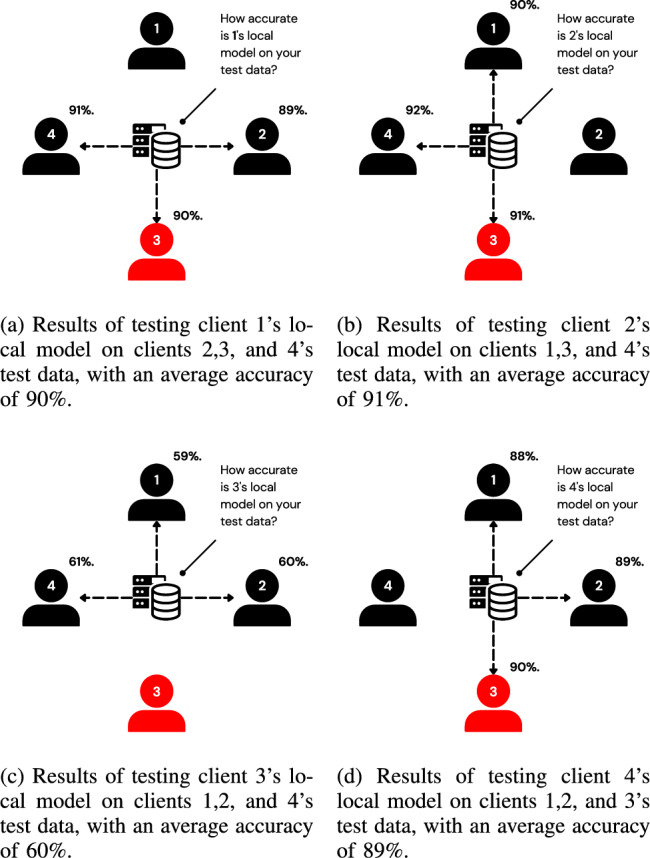
Visualization of the testing process using 4 clients.

The server needs to ask a client referred to as the tester, ”What is the accuracy of this model?” where ’this model’ refers to a local model trained by another suspected client. The suspected client is referred to as the testee, while the client testing the model is referred to as the tester. The server asks multiple testers to evaluate the same testee’s model and averages their results. This process is repeated for every client as visualized in Fig. 2.

**Fig. 3:**
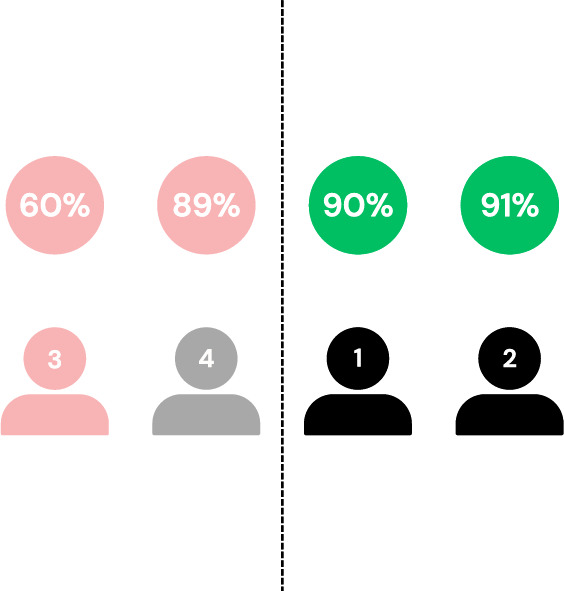
Visualization showing clients sorted by their average accuracy and filtered using the median of these values as a threshold.

The clients are then ordered based on their individual accuracies, and those falling below the *median* accuracy are isolated and excluded from aggregation, as visualized in Fig. 3. The *median* is chosen as the filtering threshold rather than the average to enhance robustness against adversarial clients. In scenarios where multiple adversaries exist, using the average can be misleading, as a few low-performing malicious clients can significantly skew the value. In contrast, the median is more resilient to outliers and better reflects the central tendency of the honest clients’ performance. This design aligns with SpyShield’s assumption that the majority of the clients are honest, as in^9,10,45^.

One issue with this approach is a vulnerability in which a tester could infer the testee’s data–analogous to revealing the location in the game Spyfall.

**Fig. 4:**
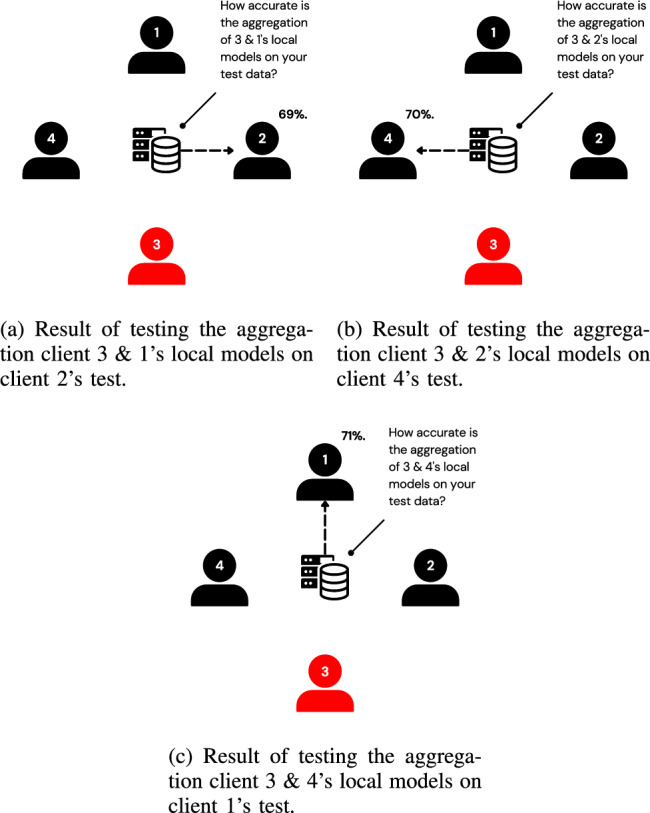
Visualization of the updated testing process on client 3, with an average accuracy of 70%.

This challenge is mitigated by aggregating the testee’s local model with those of other clients, introducing noise through this aggregation. The testee and these other clients are referred to as a ”group.” The group’s aggregated model is tested instead of the testee’s individual local model, as visualized in Fig. 4. This approach is similar to differential privacy, but instead of adding controlled noise, it disguises individual models by aggregating them with other models. As a result, no single client’s training data can be reconstructed. Furthermore, while this method does not prevent reconstruction attacks on federated learning (FL) as a whole, it ensures that the mechanism itself does not introduce vulnerabilities to such attacks.

For the remainder of the methodology, a more comprehensive discussion is provided on the nuances of SpyShield.

**Fig. 5:**
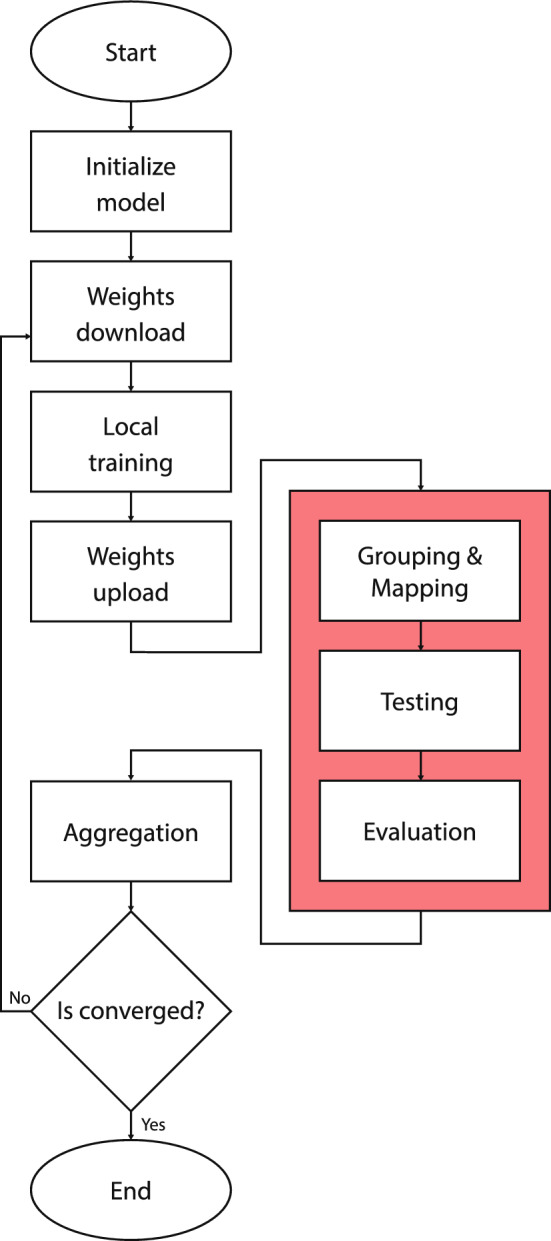
Flow chart visualization of the SpyShield -highlighted in red- in the FL pipeline.

As illustrated in Fig. 5, SpyShield can be divided into three steps:

1. Grouping and Mapping: This subsection discusses the criteria used for grouping clients and 1 assigning them to testers to ensure fairness in testing. It also details the algorithms used to enforce these criteria.
2. Aggregation and testing: This subsection discusses how a group’s local models are aggregated and how the aggregated models are sent to their respective testers to be tested.
3. Evaluation: This is the final step in SpyShield. Test results are collected from the testers and evaluated on the server to determine which clients are honest and should be included in the new global model aggregation.”

### Grouping and mapping

As discussed earlier in Subsection “Analogy”, each client’s local model is aggregated with the local models of other clients. The resulting aggregated model is then tested by another client. The clients whose local models are aggregated are collectively referred to as a group. In the example previously shown in Fig. 4, notice the following criteria:

1. The client being evaluated (client 3) is a member of all groups in the set of groups evaluating it. The client being evaluated is referred to as the testee, and the set of groups being evaluated is referred to as the grouping.
2. Each client, except the testee, belongs to one and only one group in the grouping.
3. The tester of a group must not be a member of that group.

The fact that each client, except the testee, belongs to one and only one group in the grouping mandates that the number of groups *G* in a grouping follows the equation:

1 $$\begin{aligned} G = \frac{T - 1}{c - 1}, \end{aligned}$$

where $T$ is the total number of clients and $c$ is the number of clients in a group.

The following working example of a SpyShield round with 7 clients should elaborate further.

**Fig. 6:**
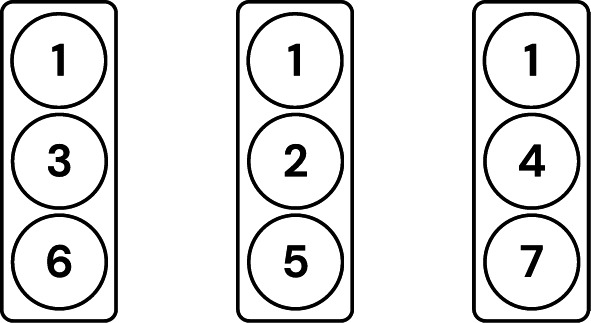
Visualization of the first testee’s grouping.

**Fig. 7:**
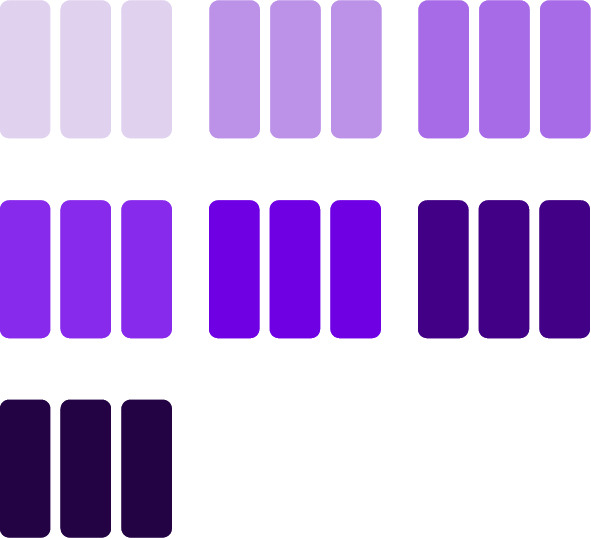
Visualization of all groupings.

First, groups are formed for each client (testee) according to the discussed criteria. Fig. 6 shows the grouping for the first testee. As per Eq. 1, the number of groups *G* is 3. Fig. 7 shows all the groups formed for each client.

**Fig. 8:**
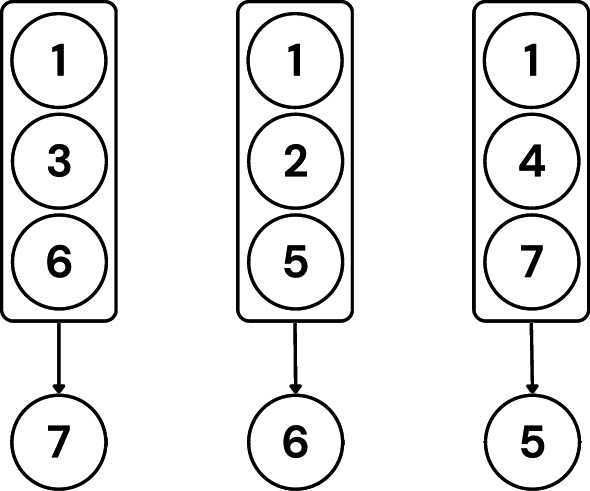
Visualization of the first testee’s group mappings.

Second, each group is mapped to a respective tester who is not a member of that group. Fig. 8 shows the first testee’s group mappings.

**Fig. 9:**
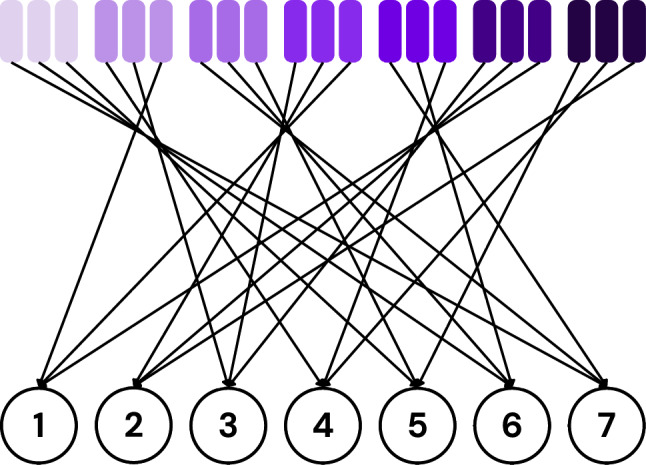
Visualization of all group mappings.

Figure 9 shows all group mappings. Notice that each client (tester) tests exactly three clients in this example. Assigning an equal number of models to each tester ensures fairness, especially during evaluation, discussed later in this section. Also, each tester tests at most one group from a grouping.

The constraints now amount to the following five constraints:

1. The client being evaluated is a member of all groups in its grouping.
2. Each client, except the testee, belongs to one and only one group in a testee’s grouping.
3. The tester of a group must not be a member of that group.
4. All clients (testers) are assigned to test an equal number of groups.
5. A tester tests at most one group from each grouping.

1) *Algorithms:* This subsection explains the algorithms used to produce the groups and mappings with the aforementioned constraints and their complexities. There are two independent algorithms: one for grouping and one for mapping.

The grouping algorithm, Algorithm 1, is a simple nested loop of time and space complexity of $O(T \times G \times c)$.

In the outer loop, for each client (testee), create a set containing all the other clients and refer to it as the remaining clients. In the inner loop for each group in the testee’s grouping, create one group by concatenating the current client with $c-1$ random clients from the set of remaining clients. Then, make sure to remove those clients from the set so that they are not included in the next group.

The time complexity of randomly selecting a client from the remaining set of clients and removing it is *O*(1), assuming a dynamic array implementation is used where one could swap the selected client with the last client in the array and remove it, since the ordering of the clients is not required.

**Algorithm 1:**
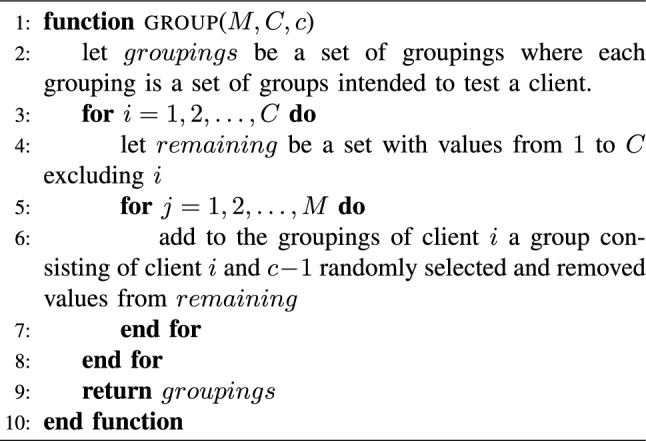
GROUPING algorithm.

The grouping Algorithm 1 guarantees meeting the first two constraints defined in Subsection "Grouping and Mapping". After the grouping algorithm is executed, the groups are mapped to their respective testers using the mapping Algorithm 2.

The mapping algorithm attempts to balance the distribution of assignments. It begins by initializing a set, referred to as frequencies, which stores tuples representing each client (tester) along with a counter tracking how many groups they have been assigned to, initially set to zero. The algorithm then iterates through the given groupings, which consist of multiple groups. For each group, it selects a client with the smallest assignment count from a subset of frequencies, ensuring that the chosen client is not already part of the group and that no other group in the same grouping is assigned to them. This approach helps distribute group assignments among clients while avoiding conflicts. Although the algorithm does not strictly guarantee perfectly equal distribution, it minimizes imbalances by always prioritizing clients with the lowest frequency.

**Algorithm 2:**
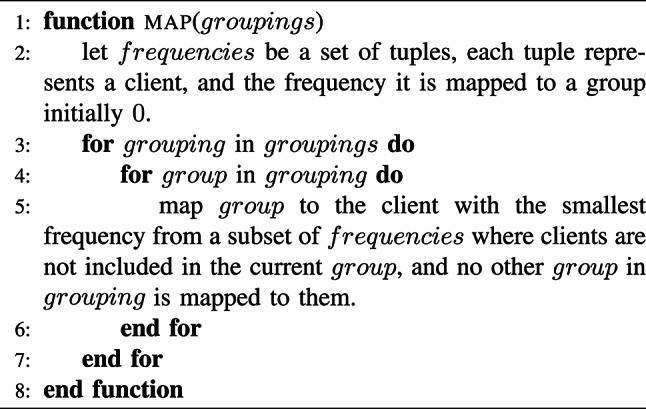
MAPPING algorithm.

Moreover, the mapping algorithm, Algorithm 2, ensures that the third and fifth constraints are always satisfied. However, the fourth constraint–ensuring that each tester is assigned an equal number of groups–is not strictly enforced. For instance, when applying this algorithm to the working example in Fig. 9, where each tester should ideally be assigned exactly three groups, the outcome may vary. Specifically, five testers might receive three groups as expected, while one tester could receive only two groups and another tester could receive four. Nevertheless, this level of imbalance is acceptable for SpyShield.

In terms of complexity, the algorithm has a space complexity of $O(T \times G)$. The time complexity varies depending on the priority queue implementation used for managing the *frequencies* set. It ranges from $O(T \times M)$ for a basic implementation to $O(T \times M \times \log (T))$ when using a binary heap or a Fibonacci heap.

### Aggregation and testing

Following the completion of the grouping and mapping phases, this section discusses the aggregation algorithm and how the aggregated models are prepared to be tested at the respective testing clients, as shown in Subsection "Grouping and Mapping".

1) *Aggregation Bias:* The aggregation of the clients’ weights in the groups is not carried out using FedAvg, as the goal of this aggregation originally is not fairness. The objective of this aggregation is to camouflage the weights of the testee client with the weights of other clients to safeguard from inferring an individual client’s training data. To ensure that the testee’s weights still meaningfully influence the test’s results, a bias towards the testee’s weights is introduced during the aggregation. This subsection elaborates on the specific implementation of this biased aggregation technique.

**Fig. 10:**
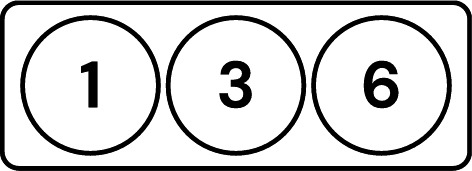
Visualization of a group with no bias, $B=1$.

**Fig. 11:**
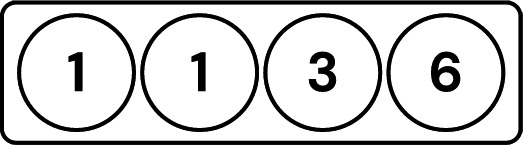
Visualization of a group with bias, $B=2$.

As presented in the working example earlier in this section, each group is constructed from 3 clients’ weights, as shown in Fig. 10, including the testee. Assume a bias of a factor of two towards the testee. This assumes that this client’s weights are included twice in the aggregation, as shown in Fig. 11. Generally, for any bias greater than zero, the weights are aggregated as follows:

2 $$\begin{aligned} W = \frac{1}{c + B - 1} (w_{1} \times B + \sum _{i=2}^{c} w_{i} ), \end{aligned}$$

where $W$ is the aggregated weights, $c$ is the number of clients being aggregated, $w_i$ are the weights of the locally trained model of the $i^{th}$ client in the group, and $B$ is the bias towards the client intended to be tested, which is the first client in the group, $w_{1}$.

**Fig. 12:**
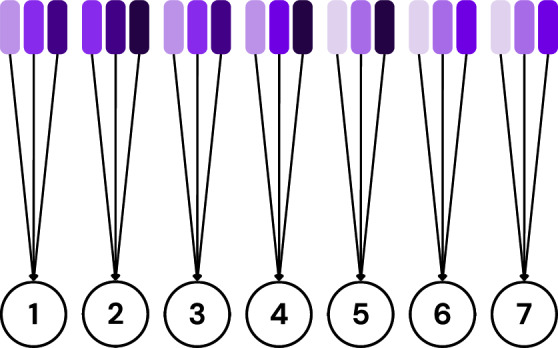
Visualization of model clustering.

2) *Model Clustering:* Once the weights of each group have been aggregated, the resulting aggregates are downloaded to their corresponding testing clients for evaluation. Prior to this, the aggregates are clustered according to the testing clients Fig. 12, enabling the server to efficiently deliver all assigned aggregates to a tester in a single batch. This streamlined approach minimizes communication overhead between the server and the participating clients, allowing SpyShield to initiate the evaluation process with a single communication round.

The second challenge SpyShield faces is that the testers themselves could be compromised as well. Moreover, if the tester is compromised, they could falsely suggest that the model they are testing is inaccurate, misleading the server into believing that the testee is the compromised one. The following subsection presents the details of evaluating the honesty of the clients as both testers and testees.

For the remainder of this work, the clients who were compromised during training are referred to as dishonest clients, while clients who were compromised during testing (during their duty as testers) are referred to as unreliable clients. This work assumes that an unreliable client is not necessarily a dishonest one and vice versa.

### Evaluation

Following testing the aggregated models on their respective testers and uploading the results to the server, the testees are evaluated to differentiate reliable testers from unreliable testers. The results of the reliable testers are then harnessed to evaluate the testees’ honesty. This subsection first discusses evaluating the reliability of the testers and then evaluates the honesty of the testees.

Referring to the example in Fig. 8, assume that client (testee) number 1 is honest and that client (tester) 7’s test data is poisoned. Therefore, assuming all other clients in the group are honest as well, the accuracy of testing the first group is below the median accuracy because of the tester’s unreliability. Now, assume that all the clients assigned to test models 7, 6, and 5 are unreliable. Then, all of the tests would result in accuracies below the median, in turn deceiving the server into unfairly flagging client (testee) number 1 as poisoned.

**Fig. 13:**
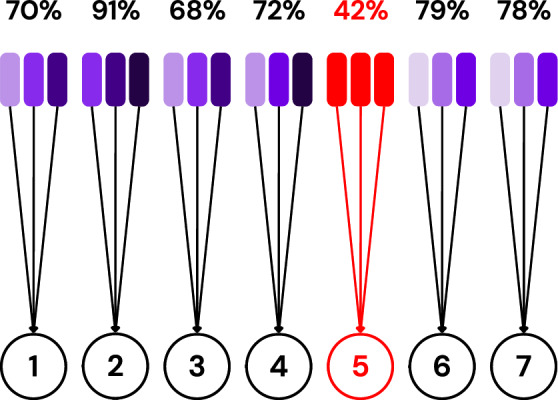
Visualization of testers’ average results in which client (tester) 5 is an unreliable tester.

To overcome this challenge, we follow the same intuition as the one we have introduced in Subsubsection “Analogy”. The server averages the results received from each tester and sorts them. Testers whose average results fall below the median are deemed unreliable, and their results are not considered when evaluating clients’ honesty. In the working example shown in Fig. 13, clients (testers) are going to be ordered as follows: 5, 3, 1, 4, 7, 6, and 5. Hence, clients 5, 3, and 1 are going to be considered unreliable, and their results won’t be used to evaluate the testees.

**Fig. 14:**
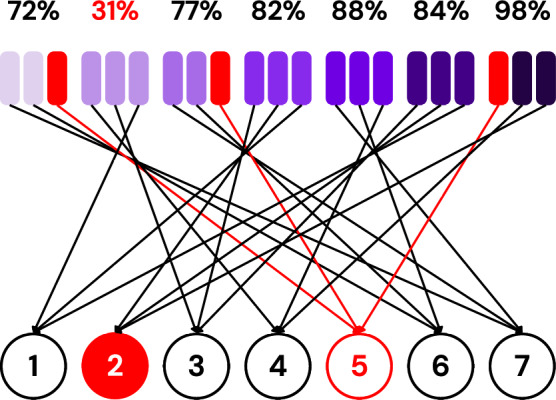
Visualization of testees’ average results.

After filtering out unreliable clients, the results received from reliable testers are grouped by testee and averaged, as shown in Fig. 14. The averages are sorted, and the testees whose average is below the median are flagged as dishonest and removed from the aggregation used to update the global model. In the working example, clients (testees) are going to be ordered as follows: 2, 1, 3, 4, 5, 6, and 7.

**Fig. 15:**
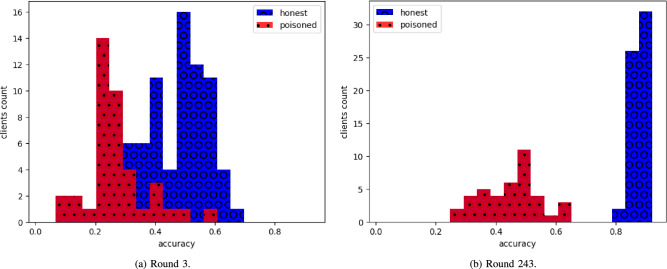
The distribution of mean accuracy per testee in a simulation with 100 clients, 40 of which are poisoned with random weights.

Observe Fig. 15, in which the average results of poisoned clients are distributed below the median.

The end output is the set of honest clients to be aggregated and used to construct the new global model used in the next run of the Federated Training round.

### Code availability

SpyShield is implemented as a single Jupyter Notebook simulation, publicly available on *GitHub* (https://doi.org/10.5281/zenodo.16785226). This single Notebook file contains the full algorithm implemented in PyTorch and everything required to run the simulation, and it was also used to generate the results in the following section.

This concludes SpyShield’s methodology. The following section discusses the experiments conducted to observe how the number of groups per client, as discussed in Subsection "Grouping and Mapping", affects SpyShield’s behavior and proves the robustness of SpyShield against poisoning attacks.

## Experiments

This section presents a comprehensive analysis of the experiments designed and executed to assess the robustness of SpyShield, the proposed defense algorithm against poisoning. The primary objective of these experiments was to evaluate SpyShield’s ability to effectively mitigate the threat of poisoning attacks, ensuring its reliability under diverse and challenging conditions. The experiments were conducted in a controlled environment, simulating real-world scenarios to obtain accurate and relevant data.

### Dataset and model

The dataset used to test SpyShield is the FashionMNIST dataset. FashionMNIST is a dataset of $28\times 28$ pixel gray-scale images of clothing items, such as shirts, sneakers, and bags, and is commonly used in federated learning poisoning experiments. It contains 70,000 examples, with 60,000 used for training and 10,000 for testing. The data is split among 150 clients using Dirichlet’s distribution with a concentration parameter $\alpha$ set to 0.5.

The FashionMNIST dataset is trained using a basic CNN image classification model composed of two convolutional layers each followed by max-pooling, a fully connected layer, and an output layer with a log-softmax activation function.

### Attack scenarios

The poisoning attacks in this study follow the experimental setup in^44^, which evaluated both data and model poisoning attacks under various poisoning ratios. That work tested random label flipping, random weights injection, and out-of-distribution attacks. Each attack was tested with three different federated learning configurations: 1-out-of-30, 5-out-of-30, and 10-out-of-50, where x-out-of-y denotes *x* malicious clients participating among *y* total clients per training round.

In our work, we adopt a similar framework with the following key differences:

- We replaced out-of-distribution attacks with cyclic label flipping, which is not part of the original study, to assess resilience against structured label manipulation that systematically shifts labels by a fixed offset.
- We adopt alternative client participation setups: 3-out-of-30, 10-out-of-50, and 40-out-of-100. These configurations allow us to assess SpyShield’s effectiveness across a broader spectrum of client population sizes and malicious participation ratios.

Moreover, the poisoning attacks covered in this work in more detail are as follows:

1. **Cyclic label flipping**, a data poisoning attack in which labels are shifted by five positions in a cyclic manner. For instance, an image initially labeled as ”6” might be relabeled as ”1.” This type of attack evaluates SpyShield’s robustness in dealing with unreliable clients since both the training and test datasets are compromised.
2. **Random label flipping**^18^, a data poisoning attack in which labels are randomly shifted forward by 1 to 9 positions in a cyclic manner. For example, a label might be moved from ”2” to ”4” or from ”8” to ”1,” depending on the random shift.
3. **Random Weights**^46^, a model poisoning attack involves a poisoned client returning a model with weights randomly generated within the range of the global model’s weights. Unlike label-based attacks, which alter the data labels, this attack disrupts the learning process by injecting randomness into the model’s parameters. By modifying the weights in a non-systematic way, this attack tests SpyShield’s ability to maintain performance despite significant deviations in model parameters from legitimate clients.

Each of these attacks is tested on three different scenarios.

1. **3-out-of-30** scenario where each FL round has 3 different clients poisoned out of 30 total clients, which makes the ratio of poisoned clients to total clients 10%.
2. **10-out-of-50** scenario where each FL round has 10 different clients poisoned out of 50 total clients, which makes the ratio of poisoned clients to total clients 20%.
3. **40-out-of-100** scenario where each FL round has 40 different poisoned clients out of 100 total clients, which makes the ratio of poisoned clients to total clients 40%.

### SpyShield’s parameters

SpyShield incorporates three parameters, two of which were introduced in the methodology section (Section “Methodology”):

1. Number of Clients per Group (*c*): The number of clients per Group has the most effect on SpyShield’s performance as it directly affects the total number of groups. Moreover, the larger, the smaller the number of clients per group, the larger the number of groups per testee. The number of groups per testee is expected to be directly proportional to SpyShield’s robustness. The experiment is conducted using two values of $c$: 3, and 4.
2. Aggregation Bias (*B*): The aggregation bias *B* is calculated based on *c* using the following equation:
  3 $$\begin{aligned} B = (c - 1) \times R, \end{aligned}$$
  where $R$ is the ratio of the multiple of the weights of the testee client in the group to the number of other clients in the group. In this work, $R$ is set to 3. Moreover, the bias by itself is not related to SpyShield’s robustness, as a bias of 6 would be significant in groups of 3 clients, but it would not be significant in groups of 10 clients. $R$ is directly related to the robustness of SpyShield; the larger the parameter $R$, the more prominent the testee’s weights effect on the test results.
3. Criterion: In the methodology section (Section “Methodology”), SpyShield relies on accuracy throughout the algorithm’s execution. However, an alternative approach could involve utilizing the loss function instead of accuracy as the primary criterion. Conceptually, this shift would not alter the core mechanics of the algorithm. In practice, rather than having testers report the accuracy of their models on the test data, they would instead return the loss values. The loss could serve as a meaningful indicator of a model’s performance. With this adjustment, the algorithm would continue to operate in the same manner but with one key modification: the selection of honest clients. Instead of identifying the top-performing clients as those with accuracy above the median, the algorithm would now select clients whose loss values fall below the median. This change in criterion would align the evaluation process with the alternative metric, as elaborated in the evaluation carried out and presented in Subsection “Evaluation”.

The three parameters discussed will be evaluated through a systematic testing process. In this study, for each scenario under consideration, SpyShield will be tested using four distinct configurations. These configurations are designed to explore the impact of varying the parameters on the algorithm’s performance and reliability. By applying SpyShield in multiple settings, this work aims to thoroughly assess how each configuration influences the effectiveness of the algorithm in detecting and mitigating potential threats. This comprehensive approach ensures that the robustness and adaptability of SpyShield are critically examined across a range of realistic conditions, thereby providing valuable insights into its practical application.

The specific configurations to be tested are outlined as follows:

1. $c$ is set to 4, $B$ is set to 9, and **accuracy** is used as the evaluation criterion.
2. $c$ is set to 4, $B$ is set to 9, and **loss** is used as the evaluation criterion.
3. $c$ is set to 3, $B$ is set to 6, and **accuracy** is used as the evaluation criterion.
4. $c$ is set to 3, $B$ is set to 6, and **loss** is used as the evaluation criterion.

These four configurations should enable one to determine how to configure SpyShield consistently in different attack scenarios for the most robust performance.

### Baseline algorithms

The SpyShield algorithm is compared with the traditional federated aggregation method, FedAvg^5^, and several leading defenses against Byzantine poisoning attacks to prove its robustness and superiority:

1. **Median**^47^ is a Byzantine-robust aggregation rule that enhances robustness against extreme values by using the median instead of the mean in the aggregation process.
2. **Trimmed-mean**^10^ represents another Byzantine-robust aggregation rule that improves robustness by using a modified mean, which involves removing a fixed percentage, *k*, of extreme values from both ends of the data distribution. In this work, the parameter *k* is set to 0.25, eliminating half of the values in total.
3. **Krum**^9^ ranks the clients based on the geometric distances of their model update distributions and selects the one closest to the majority as the aggregated model.
4. **Multi-Krum** is an extension of the original Krum algorithm, designed to further enhance robustness in Byzantine-resilient FL. It includes a parameter, $d$, which determines the number of clients to be aggregated (specifically, the first $d$ after sorting), leading to the final aggregated model. In this work, the parameter $d$ is set to 5.

### Experimental setup

In this simulation, the attack scenario is designed such that it remains unresolved: the attacker has the capability to manipulate different clients during each FL round. This means that a client who is honest in one round may become compromised in a subsequent round and vice versa. The only consistent element throughout the experiment is the fixed number of poisoned clients per round. This setup provides a more realistic representation of potential attack dynamics, as it reflects the variability and unpredictability of adversarial behavior in real-world FL systems.

The simulation was conducted for 250 rounds per run. The highest accuracy from the 250 rounds is recorded for each run. To ensure reproducibility and fairness, a fixed seed was used for sampling the dataset at the start of each simulation. Additionally, within each round, a unique seed was applied to randomly select the poisoned clients. This approach guarantees that the selection process is randomized while maintaining fairness across all baseline algorithms and SpyShield. By using unique seeds for each round, the distribution of poisoned clients remains both unpredictable and consistent, preventing bias from fixed patterns and ensuring a fair comparison of performance between different algorithms.

### Implementation details

The simulation was developed using the PyTorch library, a versatile and widely adopted Python-based framework frequently used in FL research. As noted in Section “Code Availability”, the implementation is organized as a single Jupyter Notebook, providing a complete, self-contained environment for running the algorithm, reproducing the experiments, and exploring the proposed simulation setup.

All the experiments were executed on a Windows 11 PC equipped with an NVIDIA GeForce RTX 2060 graphics card, an Intel Core i7-9750H CPU, and 16 GB DDR4 RAM.

### Results

The results from the three attack scenarios, (A) Cyclic Label Flipping, (B) Random Label Flipping, and (C) Random Weights, prove that SpyShield performs exceptionally well against various attack scenarios. As presented in Tables 1, 2, and 3, SpyShield produced models with accuracies that are very close to or even higher than that of the baseline accuracy, achieved under the no-attack scenario. This robust performance is valid against a range of numbers of malicious clients from, 3 out of 30 to more aggressive attacks of 40 out of 100.

**Table 1:** Cyclic label flipping.

	3-out-of-30	10-out-of-50	40-out-of-100
No attack	*0.8741*	*0.8772*	*0.8714*
FedAvg	0.8600	0.8294	0.8292
Krum	0.7729	0.8017	0.0774
Multi-Krum	0.8261	0.8429	0.8541
Median	0.8573	0.8620	0.8421
Trimmed Mean	0.8706	0.8593	0.8065
SpyShield ($c$ = **4**, $B$ = **9**, & criterion = **accuracy**)	0.8724	0.8656	0.8740
SpyShield ($c$ = **4**, $B$ = **9**, & criterion = **loss**)	**0.8748**	0.8678	**0.8753**
SpyShield ($c$ = **3**, $B$ = **6**, & criterion = **accuracy**)	0.8677	0.8657	0.8721
SpyShield ($c$ = **3**, $B$ = **6**, & criterion = **loss**)	0.8733	**0.8696**	0.8687

**Table 2:** Random label flipping.

	3-out-of-30	10-out-of-50	40-out-of-100
No attack	*0.8741*	*0.8772*	*0.8714*
FedAvg	0.8758	0.8471	0.8066
Krum	0.7785	0.4292	0.0839
Multi-Krum	0.8273	0.1557	0.0402
Median	0.8636	0.8545	0.8152
Trimmed Mean	0.8671	0.8647	0.8326
SpyShield ($c$ = **4**, $B$ = **9**, & criterion = **accuracy**)	0.8628	0.8656	0.8680
SpyShield ($c$ = **4**, $B$ = **9**, & criterion = **loss**)	**0.8778**	0.8655	0.8752
SpyShield ($c$ = **3**, $B$ = **6**, & criterion = **accuracy**)	0.8637	0.8677	0.8679
SpyShield ($c$ = **3**, $B$ = **6**, & criterion = **loss**)	0.8681	**0.8695**	**0.8697**

**Table 3:** Random weights.

	3-out-of-30	10-out-of-50	40-out-of-100
No attack	*0.8741*	*0.8772*	*0.8714*
FedAvg	0.5752	0.5677	0.5411
Krum	0.7769	0.7905	0.8078
Multi-Krum	0.8348	0.8411	0.8484
Median	0.8633	0.8663	0.8341
Trimmed Mean	0.8718	0.8615	0.5911
SpyShield ($c$ = **4**, $B$ = **9**, & criterion = **accuracy**)	0.7273	**0.8737**	0.6740
SpyShield ($c$ = **4**, $B$ = **9**, & criterion = **loss**)	0.6843	0.8305	**0.8776**
SpyShield ($c$ = **3**, $B$ = **6**, & criterion = **accuracy**)	**0.8805**	0.8672	0.6665
SpyShield ($c$ = **3**, $B$ = **6**, & criterion = **loss**)	0.7418	0.8698	0.8745

1) *Performance Compared to Other Defenses:* Cyclic label flipping and random label flipping are both considered to have lower invasiveness compared to Random Weights. Observe the difference between the accuracies of FedAvg without any attack and with cyclic and random label flipping. The difference does not exceed 6.5% without any defense. With these low invasiveness attacks, multiple of the benchmark defenses failed to output a model of higher accuracy than that outputted by the defenseless FedAvg, especially Krum and Multi-Krum. During the Cyclic Label Flipping 40-out-of-100 attack, Krum’s highest accuracy was 7.74%; Krum consistently selected a malicious client’s updates. This is because Krum selects updates that have the closest Euclidean distance to others. This strategy backfires in attacks that uniformly and minimally poison the updates, causing the poisoned updates to be close to each other and not significantly different from the honest updates. As the number of malicious clients increases, as in the 40-out-of-100 scenario, the Euclidean distance of the poisoned updates is lower than that of the honest updates, causing Krum to fail and start selecting poisoned updates. The same behavior is observed in Multi-Krum as well. Multi-Krum selected five poisoned updates every round of the 250 rounds during the Random Label Flipping 40-out-of-100 scenario.

The performance of Median and Trimmed Mean was comparable to SpyShield in the 3-out-of-30 and 10-out-of-50 scenarios. However, the gap between their performance and SpyShield’s widened by 5% during the 40-out-of-100 scenarios for both Cyclic and Random Label Flipping attacks. In the Random Weights 40-out-of-100 scenario, the Trimmed Mean achieved a maximum accuracy of 59.11%. Additionally, neither Median nor Trimmed Mean provided any significant advantage over FedAvg in the 3-out-of-30 Cyclic and Random Label Flipping attacks, where FedAvg’s score was slightly higher.

SpyShield proved to be the most versatile and effective against different types of attack scenarios, maintaining a performance close to the no-attack scenario even in challenging settings such as the 40-out-of-100.

2) *SpyShield Configurations Comparison:* The various configurations of SpyShield demonstrated notable differences in performance. This is caused by both the difference in $c$ (the number of clients per group) and the *criterion* (accuracy or loss).

**Fig. 16:**
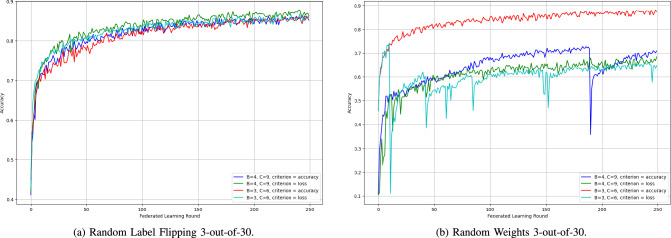
Accuracy of SpyShield configurations in random label flipping and random weights 3-out-of-30 scenarios.

**Fig. 17:**
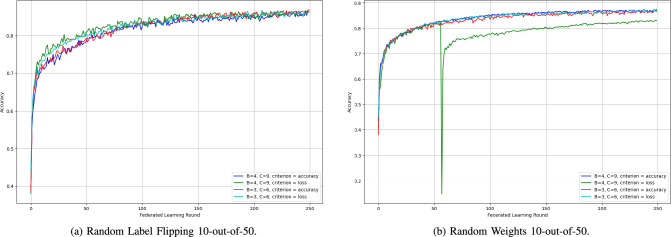
Accuracy of SpyShield configurations in random label flipping and random weights 10-out-of-50 scenarios.

**Fig. 18:**
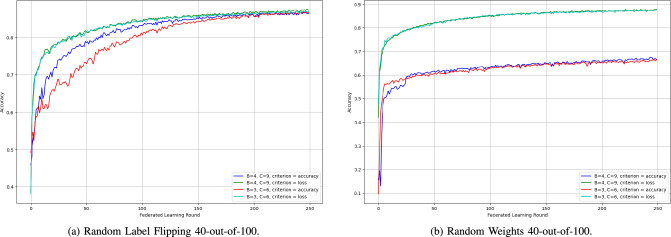
Accuracy of SpyShield configurations in random label flipping and random weights in 40-out-of-100 scenarios.

SpyShield exhibited similar behavior during both cyclic and random label-flipping scenarios. Thus, only one of the two attacks is presented in this section, Random Label Flipping as in Figs. 16, 17, and 18; the difference between the performance of the configuration was more prominent in Random Label Flipping rather than Cyclic Label Flipping.

The first variable is $c$, which is indirectly proportional to the number of groups per testee client; when set to 4, it converged slightly quicker during both attack scenarios for both accuracy and loss than when set to 3. However, both configurations converged to close accuracies. This outcome was unexpected, as the anticipated behavior was the opposite–an increased number of groups should theoretically provide more data points for analysis, leading to better performance. An exception to this pattern occurred in a single run of the simulation under the random weights attack, where the configuration with $c = 3$ not only converged faster but also achieved a final accuracy that was 15% higher.

In most cases, loss proved to be a more reliable criterion than accuracy, especially in extreme scenarios, such as the Random Weights 40-out-of-100 attack (see Fig. 18). In these runs, models evaluated using loss achieved accuracies up to 20% higher than those evaluated using accuracy. The only exception was the Random Weights 3-out-of-30 scenario, where accuracy-based evaluation resulted in a slightly higher final model accuracy.

3) *Up-scaling SpyShield to Cross-device FL:* The highest time and space complexity in SpyShield occurs during the grouping phase, as discussed in Section "Grouping and Mapping", with a complexity of $O(T \times G \times c)$. According to Equation 1, *G* is directly proportional to *T*. Therefore, in cross-device FL, where *T* is typically very large, *G* would also become large, placing a heavy computational load on the clients since each client is required to test *G* models.

However, the value computed by Equation 1 represents the maximum number of groups needed to satisfy the grouping criteria in Section "Grouping and Mapping". This maximum is not strictly required–it was simply used in the experiments to demonstrate the method under a worst-case scenario, where the highest value was 49. In practice, especially in cross-device FL settings where *G* could grow excessively, a smaller, reasonable number of groups (less than or equal to the calculated *G*) can be chosen instead. For example, using 33 groups–originally calculated for $T = 100$ and $c = 4$–can still be a reasonable choice even when $T = 1000$.

In such a scenario, the load shifts to the server, which now needs to aggregate more model updates. For instance, scaling from 100 to 1000 clients means the server must aggregate ten times more models, a reasonable overhead, considering that aggregation is the only computationally intensive operation performed at the server. This design flexibility allows SpyShield to scale effectively to large federated learning deployments.

## Conclusions

This article has introduced the SpyShield aggregation algorithm, paving a new path in the field of defense against poisoning attacks in FL. Unlike traditional approaches, SpyShield leverages client collaborations as the foundation for identifying and mitigating malicious clients. To evaluate its effectiveness, SpyShield was tested for robustness against benchmark aggregation algorithms under three types of poisoning attacks: two data poisoning attacks and one model poisoning attack. Each of these attacks was simulated in three different scenarios.

The results demonstrate that SpyShield is both robust and flexible in defending against poisoning attacks with varying levels of sophistication. Moreover, SpyShield was able to achieve the highest accuracy among the other benchmark defenses over the range of the nine different attack settings, making it a promising tool for securing FL against data and model poisoning attacks.

Moreover, the article has highlighted how SpyShield’s different configurations affect its performance, improving one’s understanding of the aggregation algorithm. However, experiments to better understand and verify the relation between the number of clients per group $c$ and the convergence rate are needed with a broader range and larger intervals.

From a broader perspective, SpyShield contributes to a generalizable direction for FL security: rather than relying solely on global statistics or central authority filtering, incorporating local client collaboration and adaptive trust mechanisms provides a scalable and effective approach to withstand a wide range of poisoning strategies.

For future research, several avenues could be explored:

1. Testing SpyShield on additional datasets: Future work could involve evaluating SpyShield on commonly used datasets such as FEMNIST and CIFAR-10 to further validate its effectiveness across diverse data types.
2. In the evaluation metrics for SpyShield, two criteria were experimented with: accuracy and loss. Each of these metrics demonstrated superiority in various tests, with accuracy excelling in some scenarios and loss proving more effective in others. By integrating both accuracy and loss into a combined evaluation, one could leverage the strengths of each criterion to improve the robustness of the SpyShield algorithm. This combination allows for a more comprehensive assessment.

Overall, SpyShield offers a versatile and collaborative defense strategy that advances the security of federated learning. It lays the groundwork for future universal solutions that dynamically adapt to attack types and learning environments. By leveraging client-side data, where critical insights for anomaly detection reside, without requiring data to leave the client.

## Supplementary Information

Supplementary Information.

## Data Availability

The datasets analyzed in this study are available from the corresponding author on reasonable request.
