# Supplementary material for: SpyShield: a Spyfall inspired defense mechanism against poisoning attacks in federated learning
Source: Sci Rep. 2025 Aug 26;15:31374. doi: 10.1038/s41598-025-16158-3 (PMC12381036; doi:10.1038/s41598-025-16158-3)
Supplement: Supplementary file 1 — Supplementary Information. [file 41598_2025_16158_MOESM1_ESM.zip › Supplementary_Material/Supplementary_Material.pdf]

I. Supplementary Material

Table I provides the working example used to explain the methodology. The table demonstrates 15 steps as follows: First, grouping the clients and then mapping them to their respective testers, where the notation $G_{i,j}$ is used to represent the jth group of client (testee) i. For example, $(G_{1,2}, 6)$ means that the second group of the first testee is mapped to be tested by client (tester) 6. Before being sent to their respective testers, the groups are clustered by tester. For example, groups $G_{2,3}, G_{4,3}, G_{6,3}$ are clustered together, as they are all mapped to tester 1. Each cluster is then sent to its respective testers, and the results are returned. The following 5 steps determine reliable testers. The average results by tester are calculated, indexed, sorted, and filtered. The remaining 6 steps determine the honest testees. The results of the reliable clients are clustered by testee. Then, the average results by testee are calculated, indexed, sorted, and filtered, finally returning the list of honest clients that can be used in the aggregation.

TABLE I: Working example.

Step	Key	Value
Grouping	groupings	$\begin{bmatrix} \{\{1,3,6\},\{1,2,5\},\{1,4,7\}\}, \\ \{\{2,4,7\},\{2,5,1\},\{2,3,6\}\}, \\ \{\{3,4,6\},\{3,2,5\},\{3,1,7\}\}, \\ \{\{4,5,7\},\{4,6,1\},\{4,3,2\}\}, \\ \{\{5,6,7\},\{5,1,2\},\{5,4,3\}\}, \\ \{\{6,1,7\},\{6,2,3\},\{6,5,4\}\}, \\ \{\{7,1,2\},\{7,3,4\},\{7,6,5\}\} \end{bmatrix}$
Mapping	mappings	$\begin{bmatrix} (G_{1,1},7),(G_{1,2},6),(G_{1,3},5), \\ (G_{2,1},4),(G_{2,2},3),(G_{2,3},1), \\ (G_{3,1},7),(G_{3,2},6),(G_{3,3},5), \\ (G_{4,1},3),(G_{4,2},2),(G_{4,3},1), \\ (G_{5,1},7),(G_{5,2},6),(G_{5,3},4), \\ (G_{6,1},3),(G_{6,2},2),(G_{6,3},1), \\ (G_{7,1},5),(G_{7,2},4),(G_{7,3},2) \end{bmatrix}$
Clustering	clusters	$\begin{bmatrix} (G_{2,3},G_{4,3},G_{6,3}), \\ (G_{4,2},G_{6,2},G_{7,3}), \\ (G_{2,2},G_{4,1},G_{6,1}), \\ (G_{2,1},G_{5,3},G_{7,2}), \\ (G_{1,3},G_{3,3},G_{7,1}), \\ (G_{1,2},G_{3,2},G_{5,2}), \\ (G_{1,1},G_{3,1},G_{5,1}) \end{bmatrix}$
Testing	results	$\begin{bmatrix} (33\%,83\%,93\%), \\ (82\%,92\%,98\%), \\ (32\%,81\%,91\%), \\ (31\%,88\%,97\%), \\ (33\%,38\%,56\%), \\ (72\%,77\%,87\%), \\ (71\%,76\%,86\%) \end{bmatrix}$
Averaging testers' results	average result per tester	$[70\%,91\%,68\%,72\%,42\%,79\%,78\%]$
Sorting testers	indexed average result per tester	$[(1,70\%),(2,91\%),(3,68\%),(4,72\%),(5,42\%),(6,79\%),(7,78\%)]$
Sorting testers	indexed sorted average result per tester	$[(5,42\%),(3,68\%),(1,70\%),(4,72\%),(7,78\%),(6,79\%),(2,91\%)]$
Filtering testers	sorted testers	$[5,3,1,4,7,6,2,]$
Filtering testers	filtered testers	$[4,7,6,2]$
Clustering results by testee	testee results	$[(72\%,71\%),(31\%),(77\%,76\%),(82\%),(88\%,87\%),(92\%,86\%),(98\%,97\%)]$
Averaging testees' results	testee results	$[72\%,31\%,77\%,82\%,88\%,84\%,98\%]$
Sorting testees	indexed average result per testee	$[(1,72\%),(2,31\%),(3,77\%),(4,82\%),(5,88\%),(6,84\%),(7,98\%)]$
Sorting testees	indexed sorted average result per testee	$[(2,31\%),(1,72\%),(3,77\%),(4,82\%),(5,88\%),(6,84\%),(7,98\%)]$
Filtering testees	sorted testees	$[2,1,3,4,5,6,7]$
Filtering testees	filtered testees	$[4,5,6,7]$
